# Addressing the challenges of cleft lip and palate research in India

**DOI:** 10.4103/0970-0358.57182

**Published:** 2009-10

**Authors:** Peter Mossey, Julian Little

**Affiliations:** Unit of Orthodontics, Dundee University Dental School, 1 Park Place, Dundee, DD1 4HR, Scotland, UK; 1 Canada Research Chair in Human Genome Epidemiology, Department of Epidemiology and Community Health, University of Ottawa, Ontario, K1H 8MB

**Keywords:** Epidemiology, Genetics, Treatment, Cleft lip, Cleft palate

## Abstract

The Indian sub-continent remains one of the most populous areas of the world with an estimated population of 1.1 billion in India alone. This yields an estimated 24.5 million births per year and the birth prevalence of clefts is somewhere between 27,000 and 33,000 clefts per year. Inequalities exist, both in access to and quality of cleft care with distinct differences in urban versus rural access and over the years the accumulation of unrepaired clefts of the lip and palate make this a significant health care problem in India. In recent years the situation has been significantly improved through the intervention of Non Governmental Organisations such as SmileTrain and Transforming Faces Worldwide participating in primary surgical repair programmes. The cause of clefts is multi factorial with both genetic and environmental input and intensive research efforts have yielded significant advances in recent years facilitated by molecular technologies in the genetic field. India has tremendous potential to contribute by virtue of improving research expertise and a population that has genetic, cultural and socio-economic diversity. In 2008, the World Health Organisation (WHO) has recognised that non-communicable diseases, including birth defects cause significant infant mortality and childhood morbidity and have included cleft lip and palate in their Global Burden of Disease (GBD) initiative. This will fuel the interest of India in birth defects registration and international efforts aimed at improving quality of care and ultimately prevention of non-syndromic clefts of the lip and palate.

## INTRODUCTION

Orofacial clefting (OFC) describes a range of abnormalities which manifest in the new born infant, OFC involves structures around the oral cavity which can extend on to the facial structures resulting in oral, facial and craniofacial deformity. The main categories are isolated cleft palate (CP) and cleft lip with or without cleft palate (CL/P). Both types may present as part of a syndrome or other associated abnormalities. Affected children have a range of functional as well as aesthetic problems. These include feeding difficulties at birth due to problems with oral seal, swallowing and nasal reguritation, hearing difficulties due to abnormalities in the palatal musculature, and speech difficulties due to nasal escape and articulation problems. These cleft defects can be surgically repaired in childhood, but residual deformity due to scarring and abnormal facial development results in continuing functional and psychosocial problems. Thus, clefts have a prolonged, adverse influence on the health and social integration of affected individuals.

### Descriptive Epidemiology: An international perspective

It is estimated that the overall global prevalence of OFC is one affected individual in every 600 new born babies. Assuming 15,000 births per hour worldwide (US Bureau of the Census, 2001), a child is born with a cleft somewhere in the world approximately every two minutes. Despite efforts to record the frequency of birth defects over the years, accurate data on the epidemiology do not exist in many countries.[1] From the available data, it may be concluded that:

- There is evidence for distinct differences between isolated CP and cleft lip with or without cleft palate CL/P.
- There is a great deal of geographical variation, more apparent for CL/P than CP.
- There is apparent variation in the proportion of OFC cases with additional congenital anomalies and syndromes.
- There is considerable international variation in the frequency of OFC, but validity and comparability of data are adversely affected by numerous factors.
- There is no consistent evidence of time trends; nor is there consistent variation by seasonality or socio-economic status.
- There are many parts of the world where little or no information on the frequency of OFCs is available, in particular in most of Africa, Central Asia, Eastern Europe, Indian sub-continent and the Middle East.

### Burden of craniofacial anomalies and cleft lip and palate in India

India is one of the many regions of the world where documentation of the rates of birth anomalies is incomplete. Reliable and complete record of statistics is difficult because of the infrastructure and due to association of craniofacial anomalies. It is known, however, that in many parts of India the parents of a child born with a cleft have no access to counseling on the care and treatment of their children. Cleft lip and palate may be perceived to be a life threatening abnormality and there may be little awareness of the fact that clefts can be surgically repaired with considerable success both aesthetically and functionally. The lack of knowledge and resources results in unacceptable delays in seeking and receiving adequate medical care, due to which, many infants with OFC die of malnutrition or infection. This grim situation is further compounded by (a) failure of healthcare authorities to recognize craniofacial anomalies as a notifiable disease, and (b) the World Health Organization (WHO) in their continuing use of the diagnostic rather than functional classification of clefts. Both these perceived problems are, however, currently being addressed.

The birth prevalence of a range of disorders which are genetic or have a genetic element in their aetiology is recorder in Table 1.

**Table 1:** Burden of Genetic Diseases at Birth in India

*Disorder*	*Birth prevalence*	*Per year*
Congenital malformations	1.00 per 50	490,000
Craniofacial anomalies	1.10 per 1000	26,950
Down syndrome	1.00 per 1139	21,510
Beta- thalassaemia	1.00 per 2700	9,074
Sickle cell disease		5,200
Metabolic diseases	1.00 per 2497	9,811

Calculated at the frequency rate obtained in the 3-center study, and at, 24.5 million births per year. Craniofacial anomalies include cases of CL/P and CP.

Three multicentric studies in India provide almost similar frequency of CFAs:

- Meta-analysis of 25 early studies from 1960 to 1979 involving 407,025 births - CL+ CP = 440 cases, 1.08 per 1000 births, CP = 95 cases, 0.23 per 1000.
- Prospective national study of malformations in 17 centers from all over India from Sept. 1989 to Sept 1990 involving 47,787 births - CL+CP = 64 cases, 1.3 per 1000 births, CP = 6 cases, 0.12 per 1000 births.
- The latest (1994-1996) three-center study involving 94,610 births in Baroda, Delhi and Mumbai – frequency of CL+CP 0.93 per 1000, and CP alone 0.17 per 1000.

Based on the last study which was most rigorously conducted, the number of infants born every year with cleft lip + cleft palate is 28,600, which means 78 affected infants are born every day, or 3 infants with clefts born every hour [Table 2].

**Table 2:** Estimated Number of Infants with common malformations

*Malformation*	*Rate per 10000*	*Total no. per year*
Neural tube defects	36.3	88,935
Talipes equinovarus	14.5	35,525
Polydactyly	11.6	28,420
Hydrocephalus alone	9.5	23,275
Cleft lip ± Cleft palate	9.3	22,785
Congenital heart disease	7.1	17,395
Hypospadias	5.0	12,250
Cleft palate alone	1.7	4,145

Calculated as in Table 1

### Registry of birth defects including CFAs in India

It is important that the issue of a birth defects registry in India, even as a pilot study, should be established to collect data in a number of centers, based upon geographic location, presence of consanguinity and high and low incidence areas as noted in previous studies. The registry should network with neonatology units in the different cities which routinely collect statistics in the newborns. In each center, a medical doctor and a social worker could work in collaboration to diagnose and collect appropriate information. Craniofacial anomalies including cleft lip and palate would be a sub-set of the data collected, and the protocols used for this should be those agreed by consensus by the WHO.[2]

## MULTIDISCIPLINARY TREATMENT, AUDIT AND RESEARCH

It is generally recognized that the optimum approach to the treatment of children born with cleft defects, either of the lip or palate, is a multidisciplinary approach. The combined efforts of a pediatrician, orthodontist, specialist nurse, cleft surgeon, speech therapist and ear, nose and throat specialist (ENT) is felt to provide the best combined expertise to ensure that the correct interventions are carried out at the appropriate time to ensure the best functional and aesthetic result.[2] The experience in different parts of the world has shown that overall there has been little improvement in care of orofacial clefts in decades and strategic co-operation between different countries to compare and contrast different treatment protocols and surgical methodologies is required to produce more consistent and predictable results.

### International collaboration in quality of care

In order to improve the quality of surgical repair as well as inputs from various specialties engaged in cleft care, international collaboration is mandatory. The success of primary surgery in the early months of life is crucial in determining for both function and esthetics; and it is in the developing world that opportunities exist for improving investigations and assimilating best treatment protocols. Currently, cleft surgery is almost completely devoid of a sound evidence base and there is a wide diversity of surgical techniques, timing and sequencing practiced throughout the world. For example, in a survey carried out through EU funding, (EUROCLEFT), 201 European cleft teams participated and 194 different surgical protocols for the repair of unilateral cleft lip and palate (UCLP) were reported.[3] The realization of this wide diversity has resulted in a multi-center collaborative effort to conduct a linked set of randomized control trials of surgery for infants with UCLP. A total of four different surgical repair techniques in terms of timing, sequencing and surgical methodology will be tested in this multi-center trial over a period of two to three years. However, since many of the outcome determinants such as speech, hearing, dento facial development and naso labial appearance will not be possible until affected children have reached the age of five, the overall duration of the study will be eight years.

Optimizing the first early surgery for infants with clefts of the lip and palate will improve physical outcomes and reduce the barrier to social integration confronted in later life. A UK national study has shown that the need for secondary corrective surgery can be 10 times higher when primary surgery is unsuccessful.[3] Identifying optimal methods of the primary surgery will reduce hospital visits during childhood and adolescence and avoid wastage of Health Services resources on outpatient therapy.

### Audit of cleft care

In any aspect of clinical medicine, quality improvement is best achieved by careful clinical audit. If the methodology for assessing outcomes can be defined and established by consensus, centers would be able to evaluate their own quality of care, compare it with other centers and enable the implementation of local quality improvement. In Europe, the first step towards attainment of minimum standards of care has been to encourage consensus on the type and timing of record collection of statistical data for the measurement of outcome. This will result in the accumulation of a wealth of data that can be used for inter-centre comparisons, both for research (i.e. establishing the best possible methods) and for audit (i.e. ensuring that the best methods are being implemented) in the future.

### Randomized controlled trials

In contemporary medicine, there is a demand for evidence based care and robust scientific enquiry is required to provide the evidence. In cleft care the diversity of surgical protocols reveals a lack of consensus on treatment methodologies and the surgeons and other members of the multi-disciplinary teams invariably believe that they are using the best methodologies available to them. It is this genuine uncertainty in conjunction with the lack of consensus that provides ethical justification for randomization and, in cleft repair, randomized control trials are justified and are now under way in Europe as part of the EUROCRAN project supported by European Commission funding.[4] European research has demonstrated that treatment protocols with low burden of care have outcomes at least as good and sometimes better than those with greater levels of intervention and greater burden of care.

## RESEARCH INTO AETIOLOGY OF OFC

Non-syndromic orofacial clefting is a polygenic, multi-factorial disorder and so both genetic and environmental factors contribute to its aetiology. The environmental factors which contribute and the genes which predispose to the condition remain obscure despite decades of research. New and innovative methods of detection of candidate genes are being applied.

Currently, optimizing treatment and surgical repair programs to rehabilitate patients in an integrated manner and prepare them for normal social life, free from prejudice and discrimination, is an important objective. However, the ultimate scientific and humanitarian objective must be primary prevention of all craniofacial abnormalities. To this end, contemporary research pursuing environmental and genetic causes is underway and is concentrating on (a) aspects of maternal medical history, lifestyle and nutrition and (b) candidate genes is under way in many parts of the world.

A recent initiative by the WHO is attempting to co-ordinate efforts throughout the world and promote international collaborative research and the mechanisms employed are detailed in a recent WHO publication entitled “Global strategies to reduce the health care burden of craniofacial anomalies”.[2]

### Environmental factors

Epidemiological and experimental evidence suggests that environmental risk factors such as maternal exposure to tobacco smoke, alcohol, poor nutrition, viral infection, medications, and teratogens in the workplace and at home in early pregnancy are important factors in aetiology. The role of maternal nutrition and, in particular, multivitamins in orofacial clefts remains unclear. Furthermore, assessment of dietary intake or biochemical measures of nutritional status are challenging and often not available in many impoverished populations with the highest rates of orofacial clefts. Socio-economic status remains an enigma, and the components of deprivation and its effects on reproductive health need further elucidation. Future studies need to measure exposures more accurately and data needs to be pooled.

The main environmental factors which have been reported as possibly increasing the risk of orofacial clefts are tobacco smoking,[5–8] alcohol consumption,[9,10] solvents[11–13,14] and agricultural chemicals.[15–17] Certain types of anti-epileptic drugs have also been reported to increase the risk.[18]

It is, however, an established fact that the magnitude of the risk of recurrence of orofacial clefts to siblings.[19,20] and the increase in risk after two or more affected siblings is greater than that predicted by the familial aggregation of environmental risk factors. If measures of genetic susceptibility are not taken into account in epidemiological studies, measures of the relative risk of a disease associated with an environmental factor can be diluted considerably.[21] In consequence, a potentially protective or terratogenic effect can be overlooked.

### Genetic factors

Orofacial clefts present as part of the phenotype in over 600 specific genetic syndromes, more commonly in association with isolated CP.[22] The proportion of CL/P associated with specific syndromes has been reported as between five and seven per cent.[23] The concordance rates for CL/P are higher in monozygotic twin pairs than in dizygotic pairs.[24–26] The familial clustering and concordance in twins of CL/P and CP has been observed to be specific for each defect, and therefore the defects are considered to be etiologically heterogeneous.[26–29] There exists a male preponderance in CL/P predominance of left-sided clefting.[30] In an attempt to determine which genes are involved, genetic linkage studies have been conducted suggesting a variety of loci, including regions on chromosomes 1, 2, 4, 6, 9, 14, 17, and 19.[31–34] and a meta-analysis of whole genome linkage studies suggests putative loci at 2q32–q35 and 9q21–q33.[35]

A variety of genetic polymorphisms have been investigated in population based association studies. Genes responsible for growth factors (e.g. TGFα, TGFβ3), transcription factors (e.g. MSX1, IRF6, TBX22), or factors which influence xenobiotic metabolism (e.g. CYP1A1, GSTM1, NAT2), nutrient metabolism (e.g. MTHFR, RARA) or immune response (e.g. PVRL1, IRF6) have been implicated. *TGFα*[36–38] and *MTHFR*[39–42] genes have been amongst the most intensively studied variants over the years. However, the results are characterized by their inconsistency, reflecting the challenges of investigating gene-disease associations and related interactions.[43]

An interesting recent finding is that the gene, *IRF6*, the gene implicated in Van der Woude syndrome (VDWS) has been shown to play a strong role in the isolated form of clefting,[44] and a number of other independent studies in a range of different populations and ethnic groups have reproduced this finding.[45–51] Other examples of gene variants involved in syndromic forms of CL/P with a Mendelian mode of inheritance producing phenocopies of non-syndromic CL/P[4] include Kallmann syndrome (FGFR1),[52] ectrodactyly-ectodermal dysplasia/clefting (TP63),[53,54] X linked ankyloglossia/clefting (TBX22),[55,56] Gorlin Syndrome (PTCH),[57] and heterozygotes for the Margarita Island clefting syndrome (PVRL1),[58] The implication is that these genes might harbour a mutation that could cause or modify the expression of isolated cleft lip and /or cleft palate.

### Gene-environment interaction

In the light of the foregoing discussion, it seems plausible that common genetic polymorphisms are modifiers of the relationship between environmental and lifestyle factors and orofacial clefts. Hence, there may be population subgroups which have a particularly high or particularly low risk of clefts due to a combination of genetic susceptibility and exposure. Genetic polymorphisms involved in the metabolism of alcohol, agents in tobacco and smoke as well as those involved in nutritional metabolism may be relevant to orofacial clefts. Hypotheses can be tested if appropriate information on these factors can be collected retrospectively from affected families.

One of the main reasons for the difficulties in determining aetiology in non-syndromic clefts is that it is polygenic multifactorial, with genetic predisposition to environmental factors being important.[59] Because of the potential public health benefits, numerous studies have been carried out to examine possible interactions that have been reported to be tested. These include those between: *TGFα* (with smoking[60–63] and vitamin supplements),[64] *TGFβ3* (with smoking, alcohol),[65–67] *MSX1* (with smoking, alcohol),[65–68] polymorphisms influencing xenobiotic metabolism (e.g. genes coding for epoxy hydrolase, glutathione-S-transferase, N-acetyltransferase) and smoking,[69–72] occupational exposures,[73] maternal medication useage,[71] retinoic acid receptor alpha (*RARA*) polymorphisms, maternal intake of vitamin A,[74] polymorphisms influencing folate metabolism (*MTHFR, RFC)* and maternal folate intake.[65,75–81]

At a WHO consensus meeting in December 2004, collaborative research pooling initiative was established through the WHO International Collaborative on Craniofacial Anomalies Project (http://www.who.int/genomics/anomalies/cfaproject/en/#mtg) to undertake meta- and pooled analyses of studies was initiated. Collaborative efforts with different populations, ethnic groups, gene pools and environmental exposures across the world will assist in determining the multiple genes that modulate the effects of an exposure.[82] The principles of genetic mendellian randomisation can be employed to aid in the identification and understanding of environmental factors in disease.[83]

## FUTURE CLEFT LIP AND PALATE RESEARCH IN INDIA

### Indian consanguinity and clefts

India has been a world leader in community genetics with the census of India, 1871 being one of the first documents to provide information on prevalence of a range of disabilities and diseases such as leprosy, blindness, deafness and insanity. India has made significant progress in combating infectious disease through improvements in sanitation, childhood nutrition, vaccination and other public health initiatives; and as a result, genetic disorders have assumed greater importance. However, the influence of consanguinous marriage has not been quantified in many diseases, with recessive genetic disorders being one example of an influence of consanguinity in the spectrum of human disease. Little is known about the influence of consanguinity on craniofacial anomalies or cleft lip and palate. Based on the *National Family and Health Survey, 1992-1993 (NFHS),*[84] consanguinous marriages are uncommon in the Northern, Eastern and North Eastern states because of the predominance of Hindu population. By comparison, in Southern India, consanguineous unions between biological kin has a long tradition.[85] The highest rates are reported in the states of Andhra Pradesh, Karnataka and Tamil Nadu, with Kerala being an exception because of the strict avoidance of consanguineous marriage amongst the large Christian population. In the pursuit of genetic research into cleft lip and palate and craniofacial anomalies, it would seem appropriate that an investigation is carried out on the influence of consanguinous marriage on non-syndromic cleft lip and palate. This may form a part of the contemplated INDIANCRAN study on cleft lip and palate, being planned in India. See below.

### Cleft lip and palate treatment research in India

Following a series of consensus meetings, among the recommendations of the WHO, , the issue of research strategies to address the significant burden of craniofacial anomalies in India were discussed. Furthermore, those delegates representing India expressed a keen desire to become involved and contribute towards quality improvement recommendations. These included the establishment of high volume treatment centers, inter-center research projects to compare outcomes, prospective registries for collection of common core outcome information and involvement in international research efforts. The large volume of cleft cases in India is contributed to partly, by the unmitigated debt of past generations wherein a proportion of the adult population with unrepaired clefts undergoes primary surgery and other rehabilitation care. To date, however, there has been little attempt to evaluate treatment outcome, carry out inter-centre comparisons of treatment protocols, evaluate residual deformity in disability or to implement quality improvement measures.

At the *Indian Society for Cleft Lip and Palate and Craniofacial Anomalies Meeting, 2006,* at Guwahati, Assam, a pilot project was carried out to begin the process of establishing baseline standards of cleft care in India and to assist Indian craniofacial treatment centers to take part in WHO initiated clinical research. This initiative was primarily aimed at the existing high volume treatment centers and to provide baseline evidence for quality of care with the possibility of comparing these results to centers within and outside of India. Ultimately the aim of such research is the improvement of quality of cleft care in India in the future. Six cleft lip and palate treatment centers in India participated in the study entitled “*Assessment of treatment outcome of cleft lip and palate surgery in non-syndromic complete unilateral cleft lip and palate patients at 5 years of age”*. Standardized study instruments were used and the outcome measures enabled inter-centre and international comparisons to be carried out. This pilot study confirmed the ability of cleft centers in India to participate in international comparisons and the results may be submitted for publication to a future issue of the *Indian Journal of Plastic Surgery*.

### The “INDIACRAN” research initiative

In order to address the challenge of both aetiology and quality of cleft care, a co-ordinated multi-center approach is being planned to address this through a project entitled the “*Indian collaboration on craniofacial anomalies*” with the acronym “INDIACRAN”. This aims to adopt a comprehensive multi-disciplinary approach and address (a) quality of care through inter-center comparisons, and (b) aetiology through a gene-environment interaction approach (described below).

- Through the auspices of a WHO Collaborating Center at the *6^th^ Asian Pacific Cleft palate Congress, Goa, 2 September 2007* a meeting *entitled “Addressing the challenge of birth defects and craniofacial anomalies in India”* was held. Through this forum the Indian Council of Medical Research (ICMR) described the initiatives and priorities in human genetics research now emerging in India. The objectives of *the Indian National Task Force on Human Genetics, which includes the development of a national database on genetic disorders, including birth defects, are as follows*.
- To establish a nationwide network of Genetic Centers capable of providing clinical and laboratory diagnosis, counselling and antenatal diagnosis, incorporating molecular techniques
- To develop a national database on genetic disorders including birth defects
- To develop trained manpower at various levels including Medical Consultants, Scientists, Information Technologists, Laboratory Technologists, Counsellors and Social Workers
- Characterization of new disease genes unique to India e.g. Handigodu disease, calcific pancreatitis, hypertrophic cardiomyopathy cardiomyopathy, etc., may include complex Polygenic disorders and genetic predisposition to cancer
- In-depth analyses of genotype phenotype correlation and study gene-gene, gene-environment interactions to understand heterogeneity of disorders
- Study genetic polymorphisms and their disease susceptibility/ drug response association in various genetically identifiable groups in India

*The emphasis and priorities of ICMR are very much in line with those of the WHO in the field of craniofacial anomalies based on the premise that different countries and different ethnic groups within the countries will have different gene pools. International collaboration is an essential component of the strategy aimed at elucidating genetic and environmental contributions to the aetiology and in due course aiming towards primary prevention*.

### Population based gene-environment interaction study

A multicenter case-parent triad / control study to investigate environmental, gene-environment, and gene-gene interactions operating in the aetiology of orofacial clefting (OFC) will be carried out. A twin track approach to the genetic investigation will be adopted:

1. Genome Wide Association Scan (GWAS)
2. Candidate genes selected *a priori*

### Genome wide association scan (GWAS)

The challenge is now to fine map the putative regions and identify genes in which variants are more likely to increase the risk for NS-OFC. Therefore, it is anticipated that there are additional genes involved in NS-OFC that are yet to be identified, and the functional effects of identified mutations have yet to be discerned. Furthermore, the genetic interaction with environmental factors will become more evident through studies evaluating maternal and foetal genotypes along with gestational environmental exposures. The triad approach being adopted will allow this to be investigated. Recent developments in high-throughput genotyping technologies and powerful statistical approaches have accelerated the discovery of loci conferring susceptibility for complex diseases through the use of genome scans. Major issues are statistical power, the value of independent replication, and the value of careful phenotyping full stop after phenolyphy. Ideally sub-phenotyping of clefts will be required, and is dependant on the recruitment of large numbers of children with clefts and their parents.. Many thousands of samples and families will be needed to unravel their contributions. Even greater numbers are required to establish definitive evidence of gene-gene and gene-environment interactions.

## CONCLUSIONS

In India the traditional unmet need in terms of primary cleft repair problem is gradually being addressed with the assistance of NGOs, and there is no shortage of surgical expertise.

- The principles of a multi-disciplinary approach to treatment have been advocated by the WHO and accepted by the craniofacial community in India.
- Some craniofacial centers in India are adopting a multi-disciplinary approach to treatment, but there remains a dearth of expertise in speech and language therapy and psychology.
- Birth defect registration and ascertainment remain significant problems and therefore no accurate figures are available on the prevalence of orofacial clefts and other craniofacial anomalies in India.
- There is an acceptance that an improvement of birth defects surveillance and research is required for (a) establishment of the birth prevalence of craniofacial anomalies, (b) improving quality of care and (c) determining the genetic and environmental aetiology of clefts in India.
- There are a number of distinctive features in the population of the Indian sub-continent that make research into both treatment and aetiology imperative; this is currently being addressed through a multi-centre project co-ordinated by WHO collaboration centres and described as the “Indiacran” research project.
